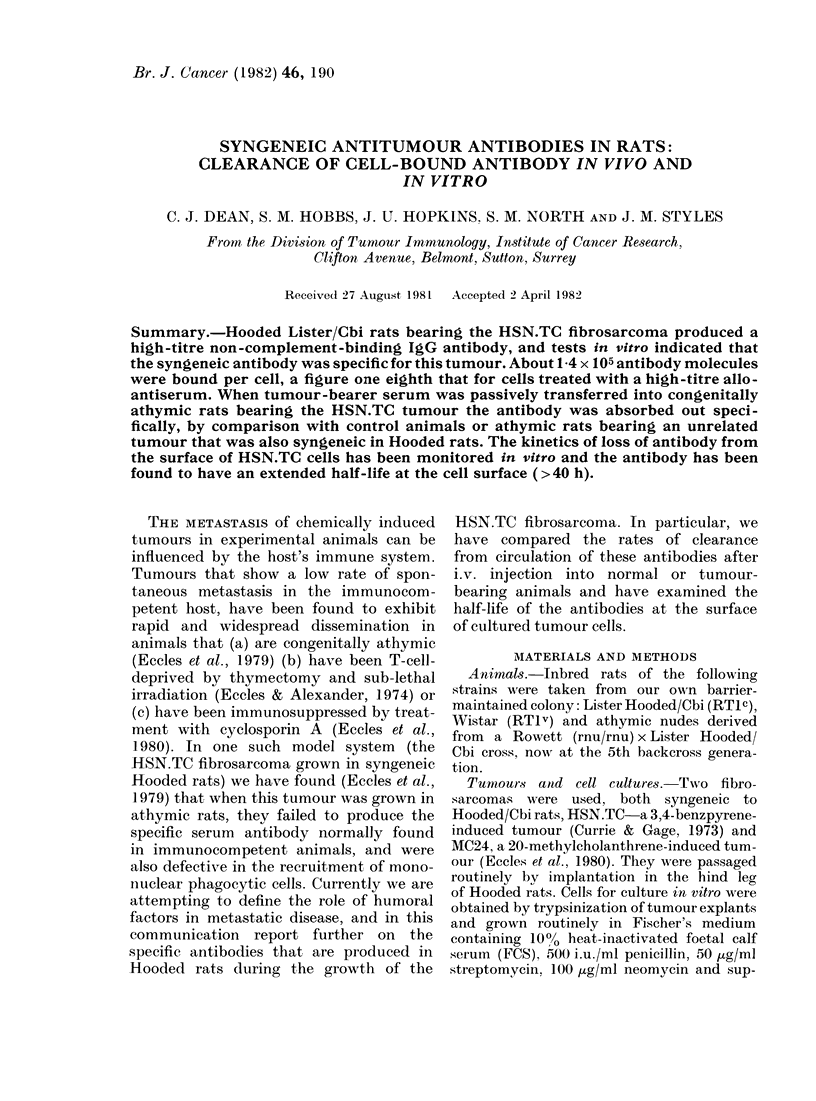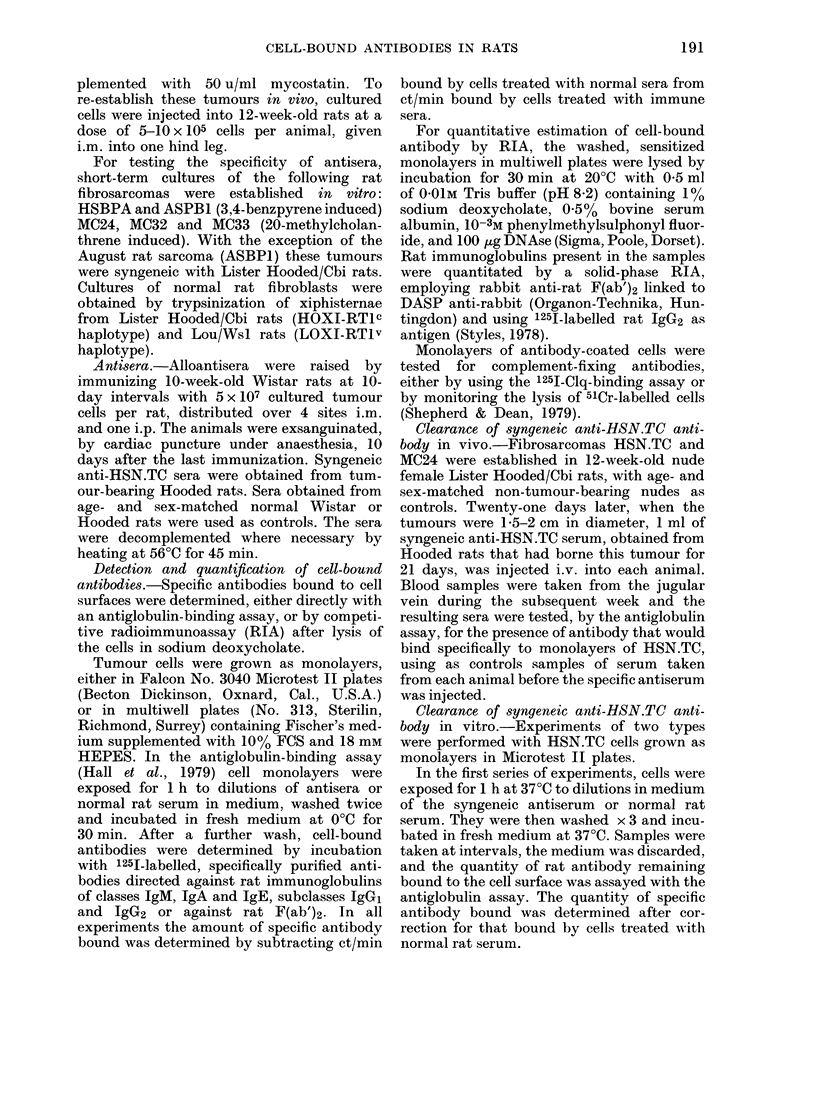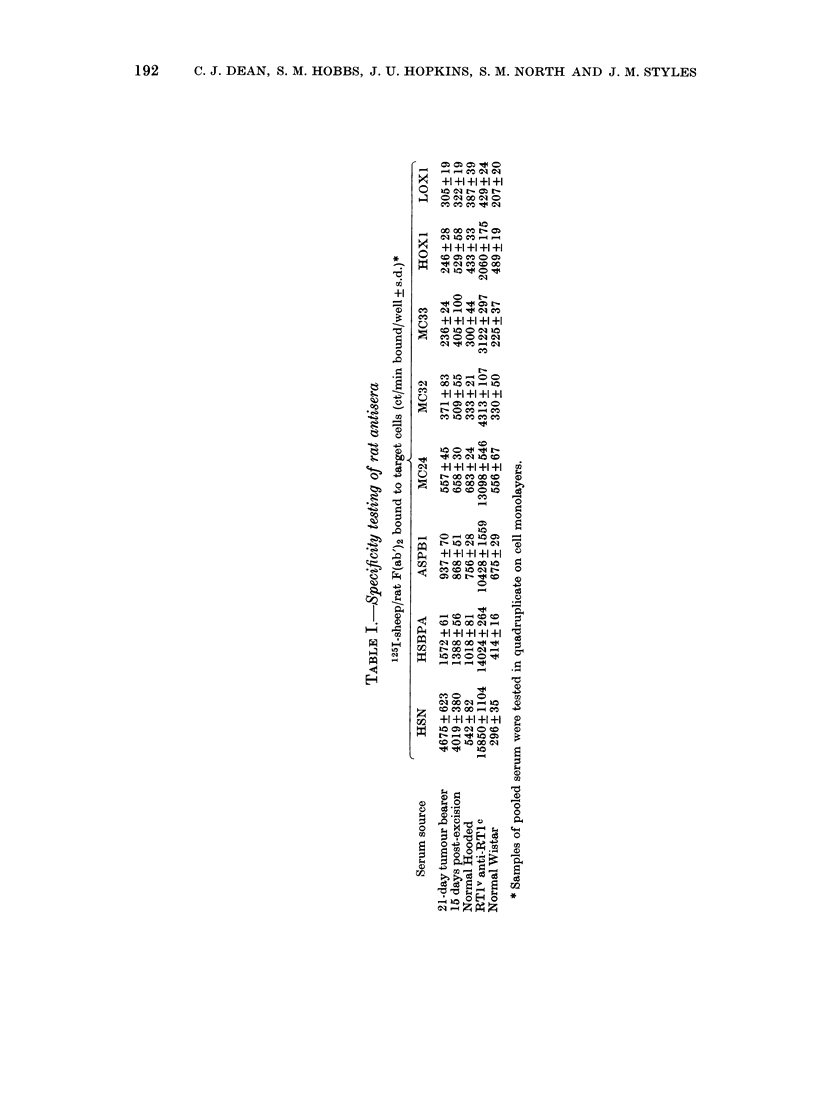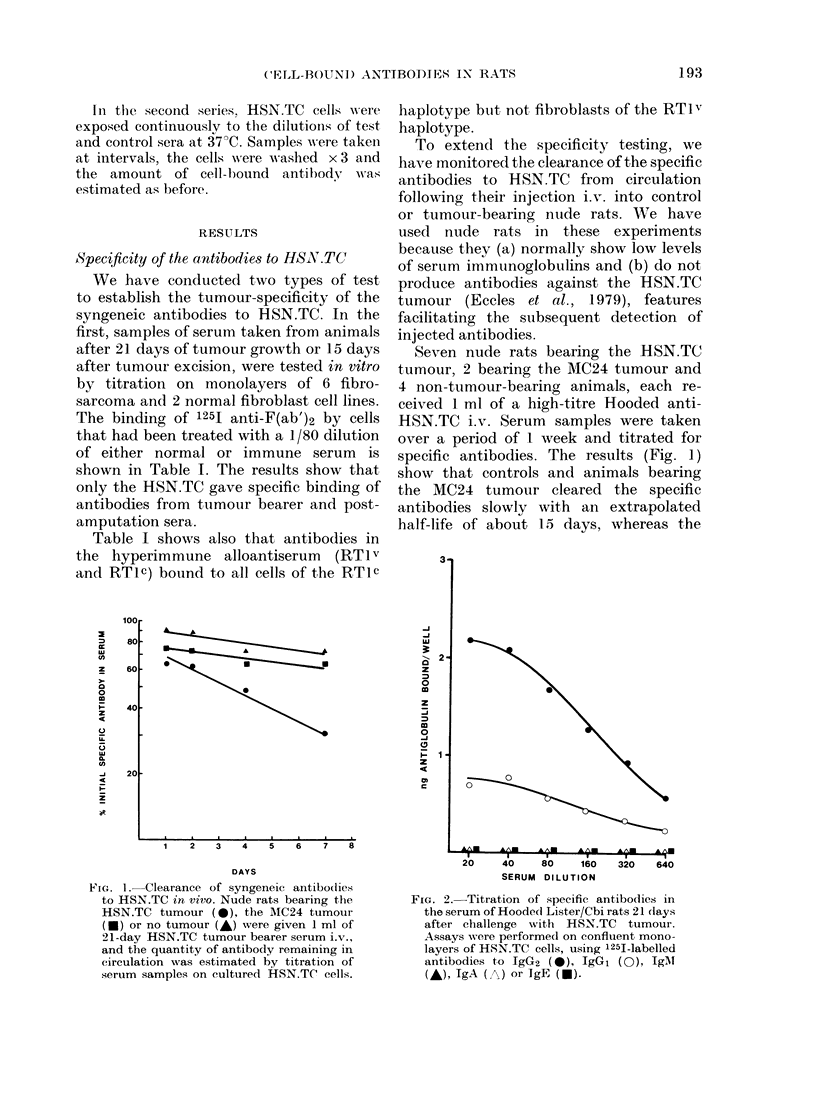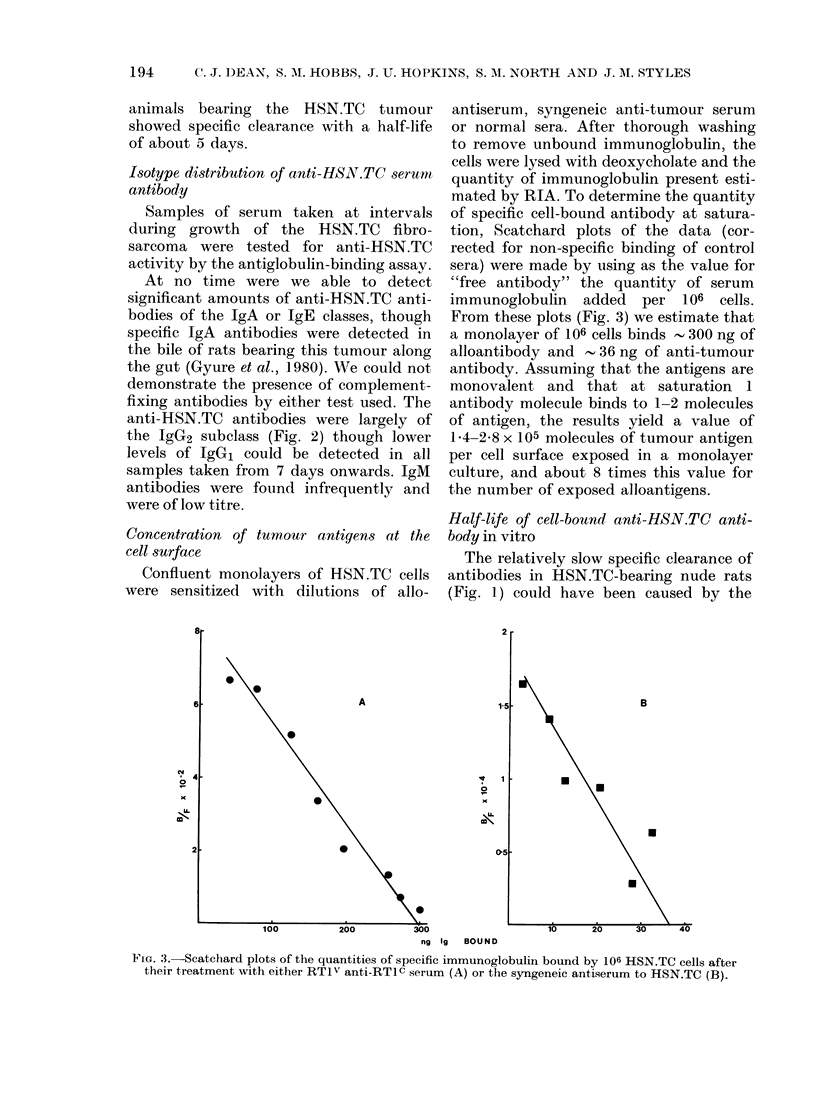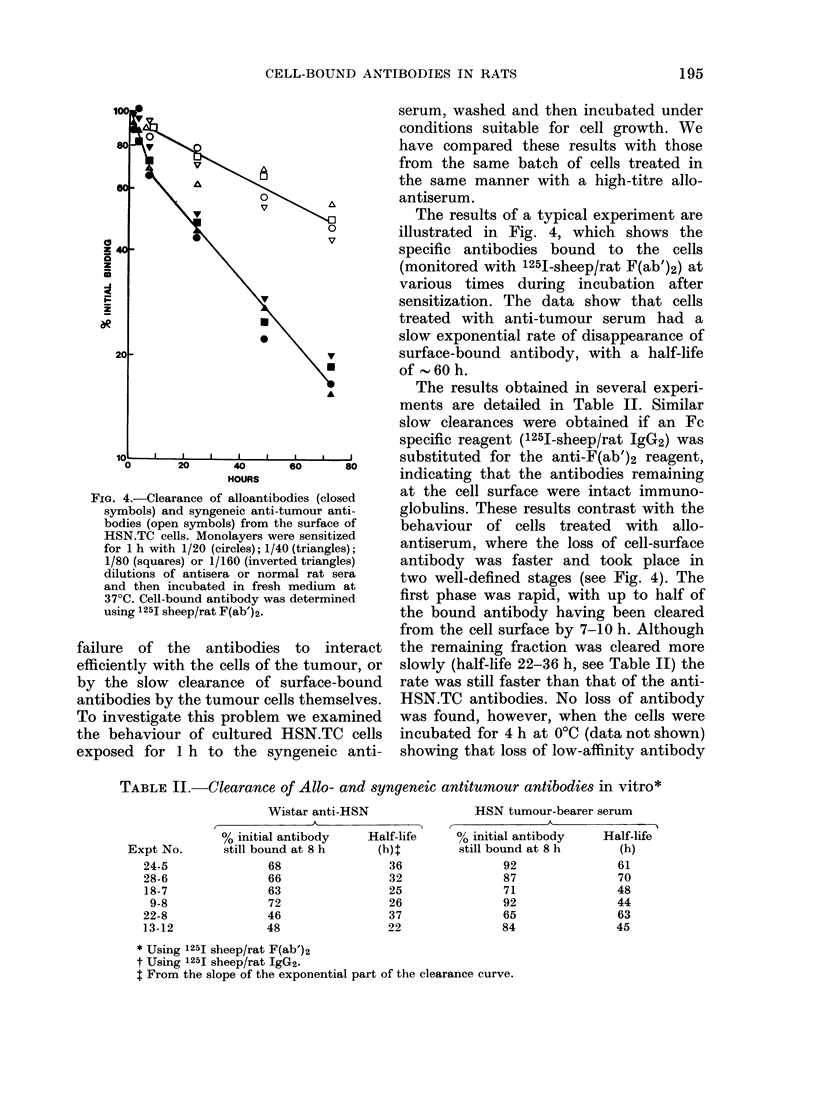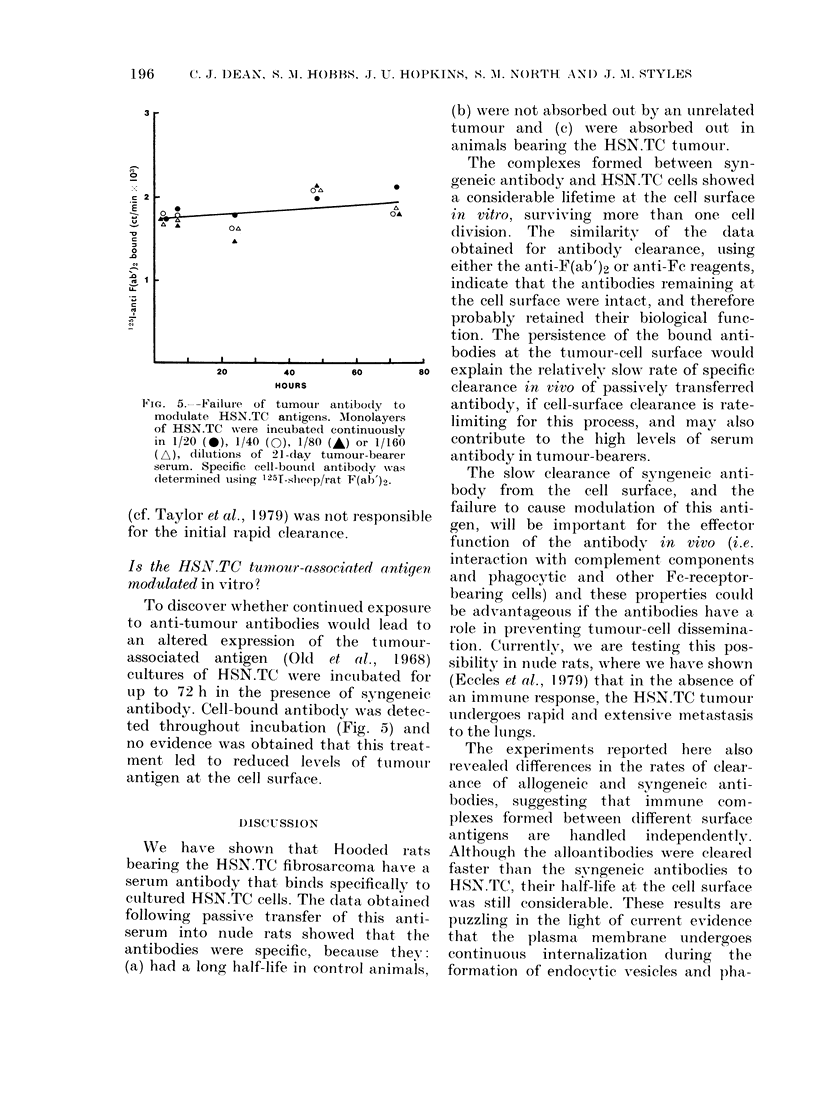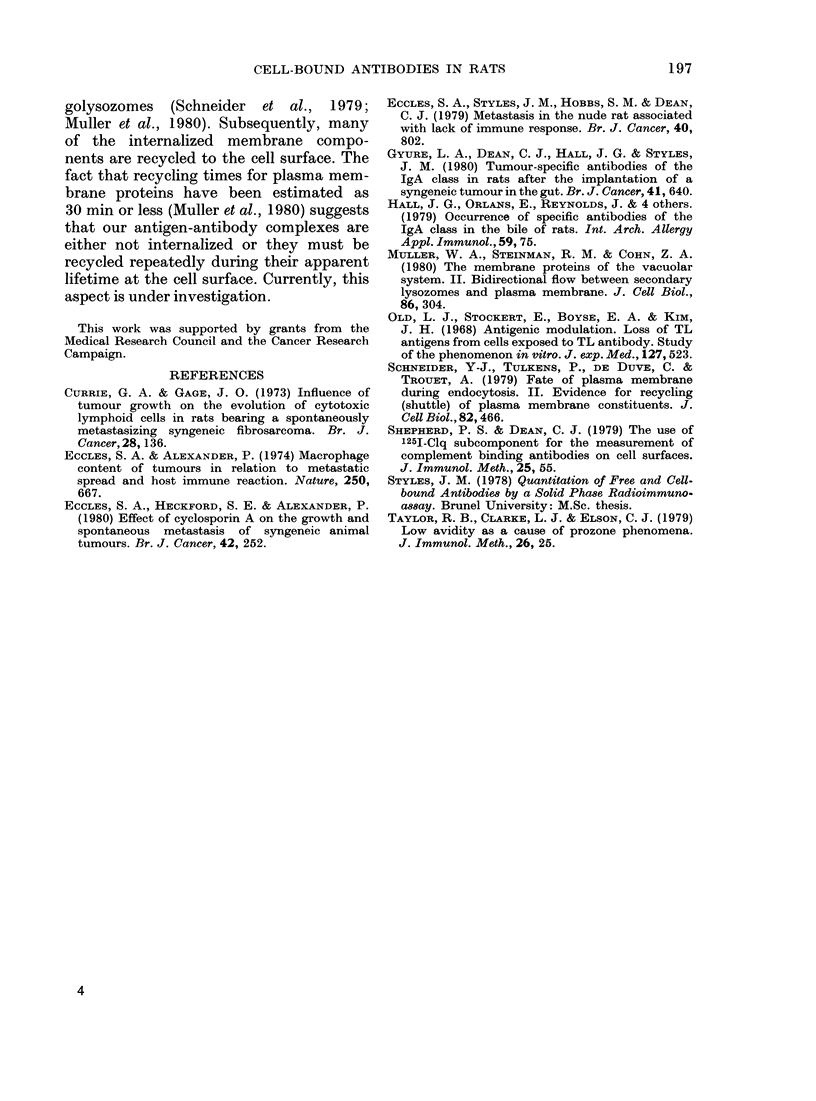# Syngeneic antitumour antibodies in rats: clearance of cell-bound antibody in vivo and in vitro.

**DOI:** 10.1038/bjc.1982.183

**Published:** 1982-08

**Authors:** C. J. Dean, S. M. Hobbs, J. U. Hopkins, S. M. North, J. M. Styles

## Abstract

Hooded Lister/Cbi rats bearing the HSN.TC fibrosarcoma produced a high-titre non-complement-binding IgG antibody, and tests in vitro indicated that the syngeneic antibody was specific for this tumour. About 1.4 X 10(5) antibody molecules were bound per cell, a figure one eighth that for cells treated with a high-titre allo-antiserum. When tumour-bearer serum was passively transferred into congenitally athymic rats bearing the HSN.TC tumour the antibody was absorbed out specifically, by comparison with control animals or athymic rats bearing an unrelated tumour that was also syngeneic in Hooded rats. The kinetics of loss of antibody from the surface of HSN.TC cells has been monitored in vitro and the antibody has been found to have an extended half-life at the cell surface (greater than 40 h).


*Br. J. Cancer* (1982) **46,** 190

### SYNGENEIC ANTITUMOUR ANTIBODIES IN RATS: CLEARANCE OF CELL-BOUND ANTIBODY *IN VIVO* AND *IN VITRO*

C. J. DEAN, S. M. HOBBS, J. U. HOPKINS, S. M. NORTH AND J. M. STYLES

*From the Division of Tumour Immunology, Institute of Cancer Research, Clifton Avenue, Belmont, Sutton, Surrey*

Received 27 August 1981 Accepted 2 April 1982

**Summary.—Hooded Lister/Cbi rats bearing the HSN.TC fibrosarcoma produced a high-titre non-complement-binding IgG antibody, and tests *in vitro* indicated that the syngeneic antibody was specific for this tumour. About $1{\cdot}4 \times 10^5$ antibody molecules were bound per cell, a figure one eighth that for cells treated with a high-titre allo-antiserum. When tumour-bearer serum was passively transferred into congenitally athymic rats bearing the HSN.TC tumour the antibody was absorbed out specifically, by comparison with control animals or athymic rats bearing an unrelated tumour that was also syngeneic in Hooded rats. The kinetics of loss of antibody from the surface of HSN.TC cells has been monitored *in vitro* and the antibody has been found to have an extended half-life at the cell surface (>40 h).**

THE METASTASIS of chemically induced tumours in experimental animals can be influenced by the host's immune system. Tumours that show a low rate of spontaneous metastasis in the immunocompetent host, have been found to exhibit rapid and widespread dissemination in animals that (a) are congenitally athymic (Eccles *et al.*, 1979) (b) have been T-cell-deprived by thymectomy and sub-lethal irradiation (Eccles & Alexander, 1974) or (c) have been immunosuppressed by treatment with cyclosporin A (Eccles *et al.*, 1980). In one such model system (the HSN.TC fibrosarcoma grown in syngeneic Hooded rats) we have found (Eccles *et al.*, 1979) that when this tumour was grown in athymic rats, they failed to produce the specific serum antibody normally found in immunocompetent animals, and were also defective in the recruitment of mononuclear phagocytic cells. Currently we are attempting to define the role of humoral factors in metastatic disease, and in this communication report further on the specific antibodies that are produced in Hooded rats during the growth of the HSN.TC fibrosarcoma. In particular, we have compared the rates of clearance from circulation of these antibodies after i.v. injection into normal or tumour-bearing animals and have examined the half-life of the antibodies at the surface of cultured tumour cells.

### MATERIALS AND METHODS

*Animals.*—Inbred rats of the following strains were taken from our own barrier-maintained colony: Lister Hooded/Cbi ($RT1^c$), Wistar ($RT1^v$) and athymic nudes derived from a Rowett (rnu/rnu) × Lister Hooded/Cbi cross, now at the 5th backcross generation.

*Tumours and cell cultures.*—Two fibrosarcomas were used, both syngeneic to Hooded/Cbi rats, HSN.TC—a 3,4-benzpyrene-induced tumour (Currie & Gage, 1973) and MC24, a 20-methylcholanthrene-induced tumour (Eccles *et al.*, 1980). They were passaged routinely by implantation in the hind leg of Hooded rats. Cells for culture *in vitro* were obtained by trypsinization of tumour explants and grown routinely in Fischer's medium containing 10% heat-inactivated foetal calf serum (FCS), 500 i.u./ml penicillin, 50 $\mu$g/ml streptomycin, 100 $\mu$g/ml neomycin and supplemented with 50 u/ml mycostatin. To re-establish these tumours *in vivo*, cultured cells were injected into 12-week-old rats at a dose of $5–10 \times 10^5$ cells per animal, given i.m. into one hind leg.

For testing the specificity of antisera, short-term cultures of the following rat fibrosarcomas were established *in vitro*: HSBPA and ASPB1 (3,4-benzpyrene induced) MC24, MC32 and MC33 (20-methylcholanthrene induced). With the exception of the August rat sarcoma (ASBP1) these tumours were syngeneic with Lister Hooded/Cbi rats. Cultures of normal rat fibroblasts were obtained by trypsinization of xiphisternae from Lister Hooded/Cbi rats (HOXI-$RT1^c$ haplotype) and Lou/Wsl rats (LOXI-$RT1^v$ haplotype).

*Antisera.*—Alloantisera were raised by immunizing 10-week-old Wistar rats at 10-day intervals with $5 \times 10^7$ cultured tumour cells per rat, distributed over 4 sites i.m. and one i.p. The animals were exsanguinated, by cardiac puncture under anaesthesia, 10 days after the last immunization. Syngeneic anti-HSN.TC sera were obtained from tumour-bearing Hooded rats. Sera obtained from age- and sex-matched normal Wistar or Hooded rats were used as controls. The sera were decomplemented where necessary by heating at 56°C for 45 min.

*Detection and quantification of cell-bound antibodies.*—Specific antibodies bound to cell surfaces were determined, either directly with an antiglobulin-binding assay, or by competitive radioimmunoassay (RIA) after lysis of the cells in sodium deoxycholate.

Tumour cells were grown as monolayers, either in Falcon No. 3040 Microtest II plates (Becton Dickinson, Oxnard, Cal., U.S.A.) or in multiwell plates (No. 313, Sterilin, Richmond, Surrey) containing Fischer's medium supplemented with 10% FCS and 18 mM HEPES. In the antiglobulin-binding assay (Hall *et al.*, 1979) cell monolayers were exposed for 1 h to dilutions of antisera or normal rat serum in medium, washed twice and incubated in fresh medium at 0°C for 30 min. After a further wash, cell-bound antibodies were determined by incubation with $^{125}$I-labelled, specifically purified antibodies directed against rat immunoglobulins of classes IgM, IgA and IgE, subclasses $IgG_1$ and $IgG_2$ or against rat $F(ab')_2$. In all experiments the amount of specific antibody bound was determined by subtracting ct/min bound by cells treated with normal sera from ct/min bound by cells treated with immune sera.

For quantitative estimation of cell-bound antibody by RIA, the washed, sensitized monolayers in multiwell plates were lysed by incubation for 30 min at 20°C with 0·5 ml of 0·01M Tris buffer (pH 8·2) containing 1% sodium deoxycholate, 0·5% bovine serum albumin, $10^{-3}$M phenylmethylsulphonyl fluoride, and 100 μg DNAse (Sigma, Poole, Dorset). Rat immunoglobulins present in the samples were quantitated by a solid-phase RIA, employing rabbit anti-rat $F(ab')_2$ linked to DASP anti-rabbit (Organon-Technika, Huntingdon) and using $^{125}$I-labelled rat $IgG_2$ as antigen (Styles, 1978).

Monolayers of antibody-coated cells were tested for complement-fixing antibodies, either by using the $^{125}$I-Clq-binding assay or by monitoring the lysis of $^{51}$Cr-labelled cells (Shepherd & Dean, 1979).

*Clearance of syngeneic anti-HSN.TC antibody* in vivo.—Fibrosarcomas HSN.TC and MC24 were established in 12-week-old nude female Lister Hooded/Cbi rats, with age- and sex-matched non-tumour-bearing nudes as controls. Twenty-one days later, when the tumours were 1·5–2 cm in diameter, 1 ml of syngeneic anti-HSN.TC serum, obtained from Hooded rats that had borne this tumour for 21 days, was injected i.v. into each animal. Blood samples were taken from the jugular vein during the subsequent week and the resulting sera were tested, by the antiglobulin assay, for the presence of antibody that would bind specifically to monolayers of HSN.TC, using as controls samples of serum taken from each animal before the specific antiserum was injected.

*Clearance of syngeneic anti-HSN.TC antibody* in vitro.—Experiments of two types were performed with HSN.TC cells grown as monolayers in Microtest II plates.

In the first series of experiments, cells were exposed for 1 h at 37°C to dilutions in medium of the syngeneic antiserum or normal rat serum. They were then washed ×3 and incubated in fresh medium at 37°C. Samples were taken at intervals, the medium was discarded, and the quantity of rat antibody remaining bound to the cell surface was assayed with the antiglobulin assay. The quantity of specific antibody bound was determined after correction for that bound by cells treated with normal rat serum.

TABLE I.—*Specificity testing of rat antisera*

| Serum source | $^{125}$I-sheep/rat F(ab')$_2$ bound to target cells (ct/min bound/well ± s.d.)* | | | | | | | |
|---|---|---|---|---|---|---|---|---|
| | HSN | HSBPA | ASPB1 | MC24 | MC32 | MC33 | HOX1 | LOX1 |
| 21-day tumour bearer | 4675 ± 623 | 1572 ± 61 | 937 ± 70 | 557 ± 45 | 371 ± 83 | 236 ± 24 | 246 ± 28 | 305 ± 19 |
| 15 days post-excision | 4019 ± 380 | 1388 ± 56 | 868 ± 51 | 658 ± 30 | 509 ± 55 | 405 ± 100 | 529 ± 58 | 322 ± 19 |
| Normal Hooded | 542 ± 82 | 1018 ± 81 | 756 ± 28 | 683 ± 24 | 333 ± 21 | 300 ± 44 | 433 ± 33 | 387 ± 39 |
| RT1$^{v}$ anti-RT1$^{c}$ | 15850 ± 1104 | 14024 ± 264 | 10428 ± 1559 | 13098 ± 546 | 4313 ± 107 | 3122 ± 297 | 2060 ± 175 | 429 ± 24 |
| Normal Wistar | 296 ± 35 | 414 ± 16 | 675 ± 29 | 556 ± 67 | 330 ± 50 | 225 ± 37 | 489 ± 19 | 207 ± 20 |

* Samples of pooled serum were tested in quadruplicate on cell monolayers.

In the second series, HSN.TC cells were exposed continuously to the dilutions of test and control sera at 37°C. Samples were taken at intervals, the cells were washed ×3 and the amount of cell-bound antibody was estimated as before.

### RESULTS

### *Specificity of the antibodies to HSN.TC*

We have conducted two types of test to establish the tumour-specificity of the syngeneic antibodies to HSN.TC. In the first, samples of serum taken from animals after 21 days of tumour growth or 15 days after tumour excision, were tested *in vitro* by titration on monolayers of 6 fibrosarcoma and 2 normal fibroblast cell lines. The binding of $^{125}$I anti-F(ab′)$_2$ by cells that had been treated with a 1/80 dilution of either normal or immune serum is shown in Table I. The results show that only the HSN.TC gave specific binding of antibodies from tumour bearer and post-amputation sera.

Table I shows also that antibodies in the hyperimmune alloantiserum (RT1$^v$ and RT1$^c$) bound to all cells of the RT1$^c$ haplotype but not fibroblasts of the RT1$^v$ haplotype.

To extend the specificity testing, we have monitored the clearance of the specific antibodies to HSN.TC from circulation following their injection i.v. into control or tumour-bearing nude rats. We have used nude rats in these experiments because they (a) normally show low levels of serum immunoglobulins and (b) do not produce antibodies against the HSN.TC tumour (Eccles *et al.*, 1979), features facilitating the subsequent detection of injected antibodies.

Seven nude rats bearing the HSN.TC tumour, 2 bearing the MC24 tumour and 4 non-tumour-bearing animals, each received 1 ml of a high-titre Hooded anti-HSN.TC i.v. Serum samples were taken over a period of 1 week and titrated for specific antibodies. The results (Fig. 1) show that controls and animals bearing the MC24 tumour cleared the specific antibodies slowly with an extrapolated half-life of about 15 days, whereas the

FIG. 1.—Clearance of syngeneic antibodies to HSN.TC *in vivo*. Nude rats bearing the HSN.TC tumour (●), the MC24 tumour (■) or no tumour (▲) were given 1 ml of 21-day HSN.TC tumour bearer serum i.v., and the quantity of antibody remaining in circulation was estimated by titration of serum samples on cultured HSN.TC cells.

FIG. 2.—Titration of specific antibodies in the serum of Hooded Lister/Cbi rats 21 days after challenge with HSN.TC tumour. Assays were performed on confluent monolayers of HSN.TC cells, using $^{125}$I-labelled antibodies to IgG$_2$ (●), IgG$_1$ (○), IgM (▲), IgA (△) or IgE (■).

animals bearing the HSN.TC tumour showed specific clearance with a half-life of about 5 days.

*Isotype distribution of anti-HSN.TC serum antibody*

Samples of serum taken at intervals during growth of the HSN.TC fibrosarcoma were tested for anti-HSN.TC activity by the antiglobulin-binding assay.

At no time were we able to detect significant amounts of anti-HSN.TC antibodies of the IgA or IgE classes, though specific IgA antibodies were detected in the bile of rats bearing this tumour along the gut (Gyure *et al.*, 1980). We could not demonstrate the presence of complement-fixing antibodies by either test used. The anti-HSN.TC antibodies were largely of the $IgG_2$ subclass (Fig. 2) though lower levels of $IgG_1$ could be detected in all samples taken from 7 days onwards. IgM antibodies were found infrequently and were of low titre.

*Concentration of tumour antigens at the cell surface*

Confluent monolayers of HSN.TC cells were sensitized with dilutions of alloantiserum, syngeneic anti-tumour serum or normal sera. After thorough washing to remove unbound immunoglobulin, the cells were lysed with deoxycholate and the quantity of immunoglobulin present estimated by RIA. To determine the quantity of specific cell-bound antibody at saturation, Scatchard plots of the data (corrected for non-specific binding of control sera) were made by using as the value for "free antibody" the quantity of serum immunoglobulin added per $10^6$ cells. From these plots (Fig. 3) we estimate that a monolayer of $10^6$ cells binds $\sim 300$ ng of alloantibody and $\sim 36$ ng of anti-tumour antibody. Assuming that the antigens are monovalent and that at saturation 1 antibody molecule binds to 1–2 molecules of antigen, the results yield a value of $1{\cdot}4$–$2{\cdot}8 \times 10^5$ molecules of tumour antigen per cell surface exposed in a monolayer culture, and about 8 times this value for the number of exposed alloantigens.

*Half-life of cell-bound anti-HSN.TC antibody* in vitro

The relatively slow specific clearance of antibodies in HSN.TC-bearing nude rats (Fig. 1) could have been caused by the

FIG. 3.—Scatchard plots of the quantities of specific immunoglobulin bound by $10^6$ HSN.TC cells after their treatment with either $RT1^V$ anti-$RT1^C$ serum (A) or the syngeneic antiserum to HSN.TC (B).

FIG. 4.—Clearance of alloantibodies (closed symbols) and syngeneic anti-tumour antibodies (open symbols) from the surface of HSN.TC cells. Monolayers were sensitized for 1 h with 1/20 (circles); 1/40 (triangles); 1/80 (squares) or 1/160 (inverted triangles) dilutions of antisera or normal rat sera and then incubated in fresh medium at 37°C. Cell-bound antibody was determined using $^{125}$I sheep/rat F(ab')$_2$.

failure of the antibodies to interact efficiently with the cells of the tumour, or by the slow clearance of surface-bound antibodies by the tumour cells themselves. To investigate this problem we examined the behaviour of cultured HSN.TC cells exposed for 1 h to the syngeneic antiserum, washed and then incubated under conditions suitable for cell growth. We have compared these results with those from the same batch of cells treated in the same manner with a high-titre alloantiserum.

The results of a typical experiment are illustrated in Fig. 4, which shows the specific antibodies bound to the cells (monitored with $^{125}$I-sheep/rat F(ab')$_2$) at various times during incubation after sensitization. The data show that cells treated with anti-tumour serum had a slow exponential rate of disappearance of surface-bound antibody, with a half-life of ~60 h.

The results obtained in several experiments are detailed in Table II. Similar slow clearances were obtained if an Fc specific reagent ($^{125}$I-sheep/rat IgG$_2$) was substituted for the anti-F(ab')$_2$ reagent, indicating that the antibodies remaining at the cell surface were intact immunoglobulins. These results contrast with the behaviour of cells treated with alloantiserum, where the loss of cell-surface antibody was faster and took place in two well-defined stages (see Fig. 4). The first phase was rapid, with up to half of the bound antibody having been cleared from the cell surface by 7–10 h. Although the remaining fraction was cleared more slowly (half-life 22–36 h, see Table II) the rate was still faster than that of the anti-HSN.TC antibodies. No loss of antibody was found, however, when the cells were incubated for 4 h at 0°C (data not shown) showing that loss of low-affinity antibody

TABLE II.—*Clearance of Allo- and syngeneic antitumour antibodies* in vitro*

| | Wistar anti-HSN | | HSN tumour-bearer serum | |
|---|---|---|---|---|
| Expt No. | % initial antibody still bound at 8 h | Half-life (h)‡ | % initial antibody still bound at 8 h | Half-life (h) |
| 24-5 | 68 | 36 | 92 | 61 |
| 28-6 | 66 | 32 | 87 | 70 |
| 18-7 | 63 | 25 | 71 | 48 |
| 9-8 | 72 | 26 | 92 | 44 |
| 22-8 | 46 | 37 | 65 | 63 |
| 13-12 | 48 | 22 | 84 | 45 |

* Using $^{125}$I sheep/rat F(ab')$_2$

† Using $^{125}$I sheep/rat IgG$_2$.

‡ From the slope of the exponential part of the clearance curve.

FIG. 5.—Failure of tumour antibody to modulate HSN.TC antigens. Monolayers of HSN.TC were incubated continuously in 1/20 (●), 1/40 (○), 1/80 (▲) or 1/160 (△), dilutions of 21-day tumour-bearer serum. Specific cell-bound antibody was determined using $^{125}$I-sheep/rat F(ab')$_2$.

(cf. Taylor *et al.*, 1979) was not responsible for the initial rapid clearance.

*Is the HSN.TC tumour-associated antigen modulated* in vitro?

To discover whether continued exposure to anti-tumour antibodies would lead to an altered expression of the tumour-associated antigen (Old *et al.*, 1968) cultures of HSN.TC were incubated for up to 72 h in the presence of syngeneic antibody. Cell-bound antibody was detected throughout incubation (Fig. 5) and no evidence was obtained that this treatment led to reduced levels of tumour antigen at the cell surface.

### DISCUSSION

We have shown that Hooded rats bearing the HSN.TC fibrosarcoma have a serum antibody that binds specifically to cultured HSN.TC cells. The data obtained following passive transfer of this antiserum into nude rats showed that the antibodies were specific, because they: (a) had a long half-life in control animals, (b) were not absorbed out by an unrelated tumour and (c) were absorbed out in animals bearing the HSN.TC tumour.

The complexes formed between syngeneic antibody and HSN.TC cells showed a considerable lifetime at the cell surface *in vitro*, surviving more than one cell division. The similarity of the data obtained for antibody clearance, using either the anti-F(ab')$_2$ or anti-Fc reagents, indicate that the antibodies remaining at the cell surface were intact, and therefore probably retained their biological function. The persistence of the bound antibodies at the tumour-cell surface would explain the relatively slow rate of specific clearance *in vivo* of passively transferred antibody, if cell-surface clearance is rate-limiting for this process, and may also contribute to the high levels of serum antibody in tumour-bearers.

The slow clearance of syngeneic antibody from the cell surface, and the failure to cause modulation of this antigen, will be important for the effector function of the antibody *in vivo* (*i.e.* interaction with complement components and phagocytic and other Fc-receptor-bearing cells) and these properties could be advantageous if the antibodies have a role in preventing tumour-cell dissemination. Currently, we are testing this possibility in nude rats, where we have shown (Eccles *et al.*, 1979) that in the absence of an immune response, the HSN.TC tumour undergoes rapid and extensive metastasis to the lungs.

The experiments reported here also revealed differences in the rates of clearance of allogeneic and syngeneic antibodies, suggesting that immune complexes formed between different surface antigens are handled independently. Although the alloantibodies were cleared faster than the syngeneic antibodies to HSN.TC, their half-life at the cell surface was still considerable. These results are puzzling in the light of current evidence that the plasma membrane undergoes continuous internalization during the formation of endocytic vesicles and phagolysozomes (Schneider *et al.*, 1979; Muller *et al.*, 1980). Subsequently, many of the internalized membrane components are recycled to the cell surface. The fact that recycling times for plasma membrane proteins have been estimated as 30 min or less (Muller *et al.*, 1980) suggests that our antigen-antibody complexes are either not internalized or they must be recycled repeatedly during their apparent lifetime at the cell surface. Currently, this aspect is under investigation.

This work was supported by grants from the Medical Research Council and the Cancer Research Campaign.

### REFERENCES

CURRIE, G. A. & GAGE, J. O. (1973) Influence of tumour growth on the evolution of cytotoxic lymphoid cells in rats bearing a spontaneously metastasizing syngeneic fibrosarcoma. *Br. J. Cancer*, **28**, 136.

ECCLES, S. A. & ALEXANDER, P. (1974) Macrophage content of tumours in relation to metastatic spread and host immune reaction. *Nature*, **250**, 667.

ECCLES, S. A., HECKFORD, S. E. & ALEXANDER, P. (1980) Effect of cyclosporin A on the growth and spontaneous metastasis of syngeneic animal tumours. *Br. J. Cancer*, **42**, 252.

ECCLES, S. A., STYLES, J. M., HOBBS, S. M. & DEAN, C. J. (1979) Metastasis in the nude rat associated with lack of immune response. *Br. J. Cancer*, **40**, 802.

GYURE, L. A., DEAN, C. J., HALL, J. G. & STYLES, J. M. (1980) Tumour-specific antibodies of the IgA class in rats after the implantation of a syngeneic tumour in the gut. *Br. J. Cancer*, **41**, 640.

HALL, J. G., ORLANS, E., REYNOLDS, J. & 4 others. (1979) Occurrence of specific antibodies of the IgA class in the bile of rats. *Int. Arch. Allergy Appl. Immunol.*, **59**, 75.

MULLER, W. A., STEINMAN, R. M. & COHN, Z. A. (1980) The membrane proteins of the vacuolar system. II. Bidirectional flow between secondary lysozomes and plasma membrane. *J. Cell Biol.*, **86**, 304.

OLD, L. J., STOCKERT, E., BOYSE, E. A. & KIM, J. H. (1968) Antigenic modulation. Loss of TL antigens from cells exposed to TL antibody. Study of the phenomenon *in vitro*. *J. exp. Med.*, **127**, 523.

SCHNEIDER, Y-J., TULKENS, P., DE DUVE, C. & TROUET, A. (1979) Fate of plasma membrane during endocytosis. II. Evidence for recycling (shuttle) of plasma membrane constituents. *J. Cell Biol.*, **82**, 466.

SHEPHERD, P. S. & DEAN, C. J. (1979) The use of $^{125}$I-Clq subcomponent for the measurement of complement binding antibodies on cell surfaces. *J. Immunol. Meth.*, **25**, 55.

STYLES, J. M. (1978) *Quantitation of Free and Cell-bound Antibodies by a Solid Phase Radioimmuno-assay*. Brunel University: M.Sc. thesis.

TAYLOR, R. B., CLARKE, L. J. & ELSON, C. J. (1979) Low avidity as a cause of prozone phenomena. *J. Immunol. Meth.*, **26**, 25.